# Expression of endogenous *Mkp1* in 6-OHDA rat models of Parkinson’s disease

**DOI:** 10.1186/2193-1801-3-205

**Published:** 2014-04-28

**Authors:** Louise M Collins, Aisling M Gavin, Sinead Walsh, Aideen M Sullivan, Sean L Wyatt, Gerard W O’Keeffe, Yvonne M Nolan, André Toulouse

**Affiliations:** Department of Anatomy and Neuroscience, University College Cork, Western Gateway Building, Cork, Ireland; Molecular Biosciences Research Division, School of Biosciences, Life Sciences Building, Museum Avenue, Cardiff, CF10 3AT UK

**Keywords:** Parkinson’s disease, 6-hydroxydopamine, MAP kinase phosphatase 1, MKP1

## Abstract

We have previously demonstrated that mitogen-activated protein kinase phosphatase 1, Mkp1, is expressed in the developing and rat adult substantia nigra and striatum, where it promotes the growth of nigral dopaminergic neurons. Mkp1 may therefore have therapeutic potential for Parkinson’s disease. In the present study, we have assessed the expression of *Mkp1* and *TH* in the substantia nigra and striatum of parkinsonian rat models. Expression was measured at 4 and 10 days post-lesion in the 6-hydroxydopamine (6-OHDA) medial forebrain bundle lesion model and after 4, 10 and 28 days in the 6-OHDA striatal lesion model. Our results show that *Mkp1* expression was transiently up-regulated in the substantia nigra at 4 days post-6-OHDA administration in the two models while *TH* expression was decreased at the later time-points examined. These data suggest that Mkp1 may play a role in counteracting the neurotoxic effects of 6-OHDA in nigral dopaminergic neurons.

## Introduction

6-hydroxydopamine (6-OHDA) is a dopamine analogue which selectively kills dopaminergic neurons and has been extensively used to model Parkinson’s disease (PD) in animals. Unilateral 6-OHDA injection in the medial forebrain bundle (MFB) of rats induces a near complete nigrostriatal degeneration and a resultant motor impairment reminiscent of that observed in Parkinson’s disease (Ungerstedt 1968; Ungerstedt and Arbuthnott 1970). However, as MFB injection of 6-OHDA results in an acute loss of dopaminergic neurons that also affects the mesocorticolimbic pathways (Breysse et al. 2007), alternative models have been developed. Specifically, four unilateral injections of 6-OHDA along the rostro-caudal axis of the striatum results in a progressive loss of approximately 80% of nigral dopaminergic neurons and motor impairments that more closely resembles the progressive nature of PD (Kirik et al. 1998).

The apparent lag between the loss of dopaminergic neurons and the appearance of motor symptoms in PD has been attributed to sprouting of remaining dopaminergic neurons to maintain striatal innervations (Hirsch 2000). Specifically, surviving dopaminergic neurons from 6-OHDA-treated animals have been shown to undergo axonal sprouting to maintain dopaminergic terminal density as a compensatory response (Stanic et al. 2003). However, the molecular mechanisms mediating this compensatory sprouting are poorly understood. We and others have recently shown that overexpression of the mitogen activated protein kinase phosphatase-1 (Mkp1) promotes the growth and branching of developing cortical (Jeanneteau et al. 2010) and midbrain dopaminergic neurons *in vitro*, and that Mkp1 expression can be modulated by 6-OHDA (Collins et al. 2013). Mitogen-activated protein kinase phosphatases (MKPs) provide a negative feedback mechanism for regulating MAPK activity (including p38, JNK and ERK) by de-phosphorylating these kinases (Farooq and Zhou 2004). Mkp1 has also been shown to offer neuroprotection in models of Huntington’s disease (HD) (Taylor et al. 2013), and to prevent neuronal death in a model of acute cerebral infarction (Koga et al. 2012). In light of these findings, the aim of the present study was to assess *Mkp1* expression in the striatum and midbrain in the 6-OHDA MFB model and the four-site 6-OHDA striatal lesion models of PD.

## Methods

### Striatal and MFB 6-OHDA lesion surgery

Date-mated Sprague–Dawley rats were provided by the Biological Services Unit, UCC. All scientific procedures were performed under a license issued by the Department of Health and Children (Ireland) and in accordance with the European Communities Council Directive (86/609/EEC). Ethical approval was granted by the Animal Experimentation Ethics Committee, UCC (AEEC). Sixty male rats (220–260 g) were used (*n* = 5 for each of saline and 6-OHDA intrastriatal injection, in groups sacrificed at 4, 10 or 28 days; *n* = 5 for each of saline and 6-OHDA MFB injection, in groups sacrificed at 4 or 10 days; *n* = 5 for intact control groups for each of the 2 experiments). 6-OHDA hydrobromide containing 0.01% ascorbic acid as a stabiliser (Sigma-Aldrich) was resuspended in saline solution and injected in test animals. Control animals were injected with the resuspension solution alone (saline). Right-sided unilateral striatal and MFB 6-OHDA lesion surgeries were previously described (Gavin et al. 2014).

### Immunohistochemistry

Serial sections (30 μm) were prepared from fixed and cryoprotected SN from 3 animals using a freezing sledge microtome. Following quenching of endogenous peroxidase activity (0.3% H_2_O_2_-10% methanol in dH_2_O) and blocking (3% normal horse or goat serum in 0.2% Triton-X-TBS), the sections were incubated overnight with primary antibodies against TH (1:1000; mouse monoclonal; Millipore) or Mkp1 (1:200; rabbit polyclonal; Santa Cruz Biotechnology) diluted in 1% normal horse or goat serum. The sections were incubated with biotinylated secondary antibodies (Vectastain® ABC Kit peroxidase mouse or rabbit IgG), followed by a streptavidin–biotin–horseradish peroxidase solution (Vector Laboratories). Immunolabelling was revealed by incubating the sections in a 0.05% solution of diaminobenzidine tetrahydrochloride-0.015% H_2_O_2_ in TBS or using the Vector Laboratories SG blue-grey. Sections were imaged on a Olympus Ax70 microscope.

### mRNA extraction and RT-QPCR

The RNA samples were obtained from Gavin et al. (2014). At 0, 4, 10 or 28 days after 6-OHDA or saline administration to the right striatum or MFB, left and right *substantia nigra* and striatum were dissected and RNA extracted and quantified. Total RNA (1 μg) was reverse-transcribed using Affinity Script reverse-transcriptase (Agilent Technologies), for 45 min at 45˚C, in a 20 μl reaction containing 5 mM dNTPs, 10 mM DTT and 10 μM random hexamer primers. 2 μl of cDNA template was used to amplify each of the normalising reference genes, glyceraldehyde 3-phosphate dehydrogenase (*Gapdh*), succinate dehydrogenase complex, subunit A (*Sdha*) and ubiquitin C (*Ubqc*), in 20 μl reactions using Brilliant III Ultra-Fast SYBR® Green QPCR Master Mix reagents (Agilent Technologies) containing 150 nM each of forward and reverse primers using conditions previously described (Gavin et al. 2014). *Gapdh*, *Sdha* and *Ubqc* amplification products were verified as accurate via melting-curve analysis of the completed PCR reaction (melting temperatures of 83.5°C, 80°C and 85°C, respectively). For *Mkp1*, 2 μl of cDNA was amplified in a 20 μl PCR reaction using Brilliant III Ultra-Fast QPCR Master Mix reagents (Agilent Technologies) containing 150 nM each of forward (5’-CTACTACAACGGTCTTCAA-3’) and reverse primers (5’-CTCTCCCAGAGTTATTGC-3’) and 300 nM of a FAM/BHQ1 dual-labelled hybridization probe (5’-FAM-TTCCCTGTCTCCATCCCTGT-BHQ1-3’, Eurofins). For *TH* amplification, previously described conditions, were used (Gavin et al. 2014). The initial quantities of cDNA in each PCR reaction were determined by comparison to standard curves that were generated by serial dilutions of reverse-transcribed RNA purified from either the striatum or SN. Values for each gene of interest were normalised to a geometrical mean of the three reference genes. Each reaction was repeated 3 times and amplifications were performed in triplicate each time.

### Statistical analysis

Each data point represents grouped data from 3 separate RNA preparations, taken from 4 animals. One-way ANOVA with *post*-*hoc* Tukey’s test were used to determine significance. Data are expressed as mean with standard errors and deemed significant when *p* <0.05.

## Results

### Mkp1 is expressed in adult rat SN dopaminergic neurons

**Figure 1:**
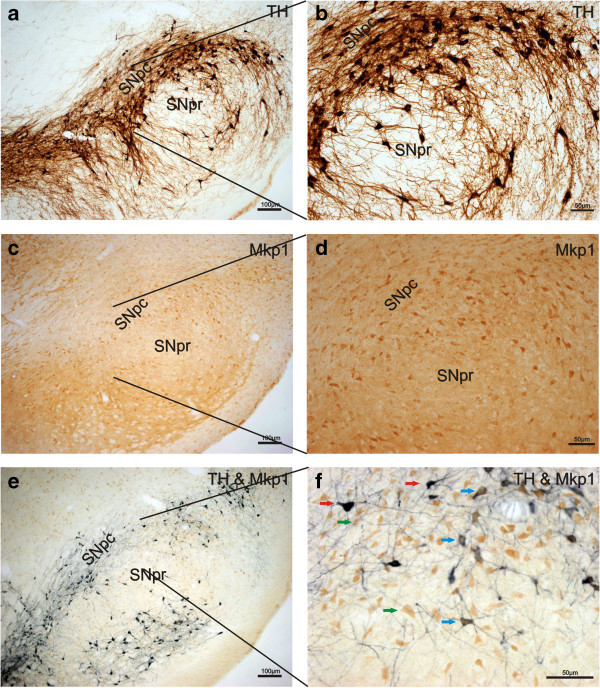
**Mkp1 is expressed in dopaminergic neurons in the adult rat SN.** Representative photomicrograph showing **(a, b)** TH expression (brown), **(c, d)** Mkp1 expression (brown) and **(e, f)** Mkp1 expression (brown) in TH^+^ (blue/black) dopaminergic neurons throughout the adult rat SN under basal conditions. **(f)** Co-localisation in the SNpc. Red arrows indicate TH^+^ (blue/black), green arrows indicate Mkp1^+^ (brown) cells while the blue arrow indicates double labelling of Mkp1^+^/TH^+^ cells. Scale bars =100 μm **(a, c, e)** or 50 μm **(b, d, f)**.

We have previously demonstrated that Mkp1 is expressed in the developing midbrain, including dopaminergic neurons, and that it is also expressed in the adult SN (Collins et al. 2013). In order to further characterize the adult expression of Mkp1, particularly in dopaminergic neurons, we performed an immunohistochemistry for TH and Mkp1 in the adult SN. Representative images in Figure 1 show that TH^+^ (Figure 1a,b) and Mkp1^+^ cells (Figure 1c,d) were evident in the adult rat SNpc and SNpr under basal conditions. Double-labelling experiments revealed that TH^+^ dopaminergic neurons express Mkp1 (Figure 1e,f) indicating a potential regulatory role for Mkp1 in these cells. In particular, Mkp1 expression was prominent in the soma rather than in the neurites of these cells (Figure 1f). Mkp1 is more broadly expressed, and is also present in TH- cells (Figure 1f). Negative controls where primary antibodies were omitted showed no evidence of staining (data not shown). This suggests that even though some dopaminergic neurons will degenerate following the 6-OHDA injection, it is possible to detect Mkp1 mRNA expression from surrounding cells.

### Mkp1 mRNA expression is transiently increased in the SN following unilateral 6-OHDA MFB or striatal lesions

**Figure 2:**
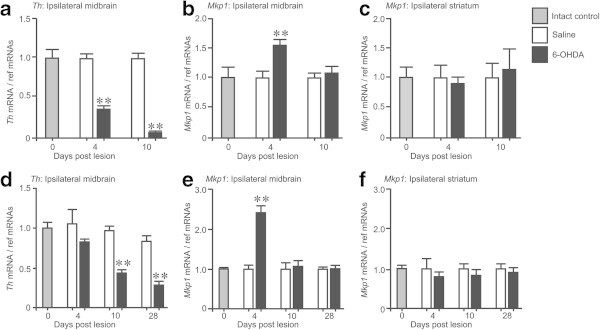
***Mkp1*** **mRNA expression is transiently increased in the SN following unilateral 6**-**OHDA MFB or striatal lesions.** qRT-PCR of **(a)** *Th*, **(b)** *Mkp1* mRNA in the ipsilateral SN and **(c)** *Mkp1* mRNA in the ipsilateral striatum at 0, 4, and 10 days after administration of 6-OHDA or saline into the right MFB. qRT-PCR of **(d)** *Th*, **(e)** *Mkp1* mRNA in the ipsilateral SN and **(f)** *Mkp1* mRNA in the ipsilateral striatum at 0, 4, 10 and 28 days after administration of 6-OHDA or saline into the right striatum. ** *p* < 0.01 vs. saline-treated control at the same time-point; one-way ANOVA with *post*-*hoc* Tukey’s test (n = 4 animals). Data are expressed as mean with standard errors.

The 6-OHDA-induced lesions resulted in a significant ipsilateral dopaminergic neuronal depletion accompanied by increased contralateral amphetamine-induced rotations (Gavin et al. 2014). To analyse the temporal dynamics of dopaminergic neuronal loss in both the MFB and striatal 6-OHDA lesion models, *Th* mRNA expression was quantified in the SN at 0, 4 and 10 days post-6-OHDA MFB lesion (Figure 2a) or at 0, 4, 10, and 28 days post-6-OHDA striatal lesion (Figure 2d, data from Gavin et al., 2014). Our results demonstrated that right-sided unilateral 6-OHDA injections to the right MFB resulted in a reduction in *Th* mRNA expression in the ipsilateral SN at 4 and 10 days post-lesion compared to saline-treated animals at the same time point (*p* < 0.01; Figure 2a). Interestingly, *Mkp1* mRNA expression increased at 4 days post-lesion in the ipsilateral SN compared to saline-treated animals at the same time point (*p* < 0.01; Figure 2b) and returned to baseline by 10 days post-lesion (Figure 2b). There was no significant change detected in *Mkp1* mRNA expression in the ipsilateral (Figure 2c) striatum after 6-OHDA administration to the MFB compared to saline-treated animals at the same time point.

Unilateral 6-OHDA injections to the right striatum resulted in a reduction in *Th* mRNA expression in the ipsilateral SN of these animals at 10 and 28 days post-lesion compared to saline-treated animals at the same time point (*p* < 0.01; Figure 2d). We observed no reduction in *Th* mRNA expression at 4 days post-lesion (Figure 2d) unlike that observed in the MFB model (Figure 2a). There was an increase in *Mkp1* expression at 4 days post-lesion compared to saline-treated animals at the same time point (*p* < 0.01; Figure 2e). This increase returned to baseline by 10 days post-lesion (Figure 2e). There was no change detected in *Mkp1* mRNA expression in the ipsilateral (Figure 2f) striatum after 6-OHDA administration to the striatum compared to saline-treated animals at the same time point.

## Discussion

This study examined *Mkp1* mRNA expression in the SN and striatum of the adult rat nigrostriatal pathway in the MFB and striatal 6-OHDA lesion models of PD. It has been reported that these lesion paradigms lead to different spatiotemporal patterns of nigrostriatal cell body and terminal degeneration. In terms of the dopaminergic cell bodies in the SN, both the striatal and MFB lesion have been shown to induce neuronal loss that is first apparent at 72 h post-lesioning and worsens thereafter (Walsh et al. 2011). The MFB lesion paradigm is generally recognised as a more severe phenotype representing the end stages of PD with near complete neuronal depletion in the SN while the striatal model leads to a more subtle and progressive degeneration reminiscent of the early stages of the disease (Bove and Perier 2012). This is in general agreement with our quantitative PCR data showing a ~60% reduction in *Th* mRNA in the MFB lesion model and a non-significant 20% reduction in the striatal lesion model at 4 days post-lesion becoming more pronounced after 10 and 28 days. In the current study, we report an up-regulation of *Mkp1* mRNA expression in the SN in both the MFB and the striatal 6-OHDA lesion models at 4 days post-lesion, which returned to baseline by 10 days post-lesion. There was no up-regulation of *Mkp1* in the contralateral striatum or SN (data not shown), or in the ipsilateral striatum, indicating that the up-regulation of *Mkp1* mRNA was specific to the 6-OHDA-induced changes in the SN. As *Mkp1* mRNA is increased at 4 days post-lesion, which precedes the degeneration of dopaminergic neurons at day 10 in the striatal model, it is plausible that the progressive degeneration in this model may allow a window of opportunity for Mkp1 to attempt to rescue dopaminergic nerve terminals. The neurodegeneration observed in the MFB model is of greater magnitude and is usually accepted as occurring at earlier time-points following 6-OHDA infusion. Considering the 50% increase in Mkp1 expression observed in a severely depleted SN, it is possible that this increase represents the tissue’s attempt to prevent neurodegeneration. We cannot rule out the possibility that changes in *Mkp1* expression are provided by other cell types, as *Mkp1* has previously been shown to be up-regulated in microglia after treatment with lipopolysaccharide, albeit in cortical tissue (Camps et al. 2000). We have previously shown that 6-OHDA up-regulates Mkp1 protein expression in embryonic ventral mesencephalon samples while it is specifically down-regulated in 6-OHDA-treated dopaminergic neurons cultured *in vitro* (Collins et al. 2013), suggesting that up-regulation of *Mkp1* expression detected in the present study may occur mostly in glial cells. It is also tempting to speculate that *Mkp1* may be up-regulated in both SN dopaminergic neurons and microglia in an attempt at cellular neuroprotection following exposure to 6-OHDA. Indeed, strategies that increase Mkp1 in the central nervous system (CNS) have been shown to be neuroprotective in a number of animal models of neurodegenerative and inflammatory CNS insults (Eljaschewitsch et al. 2006; Koga et al. 2012; Taylor et al. 2013).

The molecular mechanisms that lead to the induction of *Mkp1* following 6-OHDA insult are unclear, however it may result from a MAPK-Mkp feedback loop (Camps et al. 2000). Specifically, it has been previously demonstrated that unilateral administration of 6-OHDA to the MFB resulted in a significant and sustained increase in the level of phospho-p38 in dopaminergic neurons in the ipsilateral SN for at least 10 days post-lesion (Crotty et al. 2008). Mkp1 forms a negative feedback loop to control p38 activity, and consequently the expression of *Mkp1* is induced by stimuli that activate MAPK pathways, including p38 (Camps et al. 2000). Given that 6-OHDA induces phospho-p38 expression in dopaminergic neurons *in vitro* (Collins et al. 2013) and *in vivo* (Crotty et al. 2008), it is possible that this also is accompanied by an induction of *Mkp1* in an attempt to down-regulate p38 activation. In rodents, this compensatory mechanism may be, at least in part, the reason that striatal dopaminergic terminal density is maintained at normal levels until >70% of nigral cells are lost (Finkelstein et al. 2000; Stanic et al. 2003). Thus, Mkp1, through its regulation of p38 activation, may be critically involved in inducing or maintaining this compensatory mechanism.

This study demonstrates that the expression of *Mkp1* mRNA is regulated in a precise spatio-temporal pattern following 6-OHDA insult to the MFB and/or striatum. These results raise the possibility that the sprouting of dopaminergic nerve terminals in the striatum following exposure to 6-OHDA may be mediated by an up-regulation of *Mkp1*. In the wider context of PD, these data suggest that up-regulation of *Mkp1* in the parkinsonian SN may occur at early stages of the disease in an attempt at endogenous neuroprotection, but that at later stages of the disease, when these *Mkp1* inductive mechanisms fail, a more rapid degeneration of dopaminergic neurons occurs. Therefore, it will be important to study the relative levels of *Mkp1* at different stages of PD, as strategies aimed at augmenting *Mkp1* expression may be therapeutically beneficial.

## Abbreviations

CNS: Central nervous system
HD: Huntington’s disease
MFB: Medial forebrain bundle
PD: Parkinson’s disease, SN, Substantia nigra
SNpc: Substantia nigra pars compacta
SNpr: Substantia nigra pars reticulate.
